# Survival, Growth, and Reproduction: Comparison of Marbled Crayfish with Four Prominent Crayfish Invaders

**DOI:** 10.3390/biology10050422

**Published:** 2021-05-10

**Authors:** Antonín Kouba, Boris Lipták, Jan Kubec, Martin Bláha, Lukáš Veselý, Phillip J. Haubrock, Francisco J. Oficialdegui, Hamid Niksirat, Jiří Patoka, Miloš Buřič

**Affiliations:** 1 South Bohemian Research Center of Aquaculture and Biodiversity of Hydrocenoses, Faculty of Fisheries and Protection of Waters, University of South Bohemia in České Budějovice, Zátiší 728/II, 38925 Vodňany, Czech Republic; liptaq.b@gmail.com (B.L.); kubecj@frov.jcu.cz (J.K.); blaha@frov.jcu.cz (M.B.); veselyl@frov.jcu.cz (L.V.); phillip.haubrock@senckenberg.de (P.J.H.); niksirat@frov.jcu.cz (H.N.); buric@frov.jcu.cz (M.B.); 2 Department of River Ecology and Conservation, Senckenberg Research Institute and Natural History Museum Frankfurt, Clamecystrasse 12, 63571 Gelnhausen, Germany; 3 Department of Wetland Ecology, Doñana Biological Station (EBD-CSIC), C/Américo Vespucio 26, Isla de la Cartuja, 41092 Seville, Spain; oficialdegui@ebd.csic.es; 4 Department of Zoology and Fisheries, Faculty of Agrobiology, Food and Natural Resources, Czech University of Life Sciences Prague, Kamýcká 129, 16500 Prague-Suchdol, Czech Republic; patoka@af.czu.cz

**Keywords:** biological invasion, pet trade, animal release, species interactions, sympatry

## Abstract

**Simple Summary:**

Biological invasions exert tremendous impacts on native biodiversity and ecosystem functioning. Invasive crayfish species are well known for their particularly vigorous impacts. Recent research indicated that locations with multiple invasive crayfish species are increasing, yet questions asking which species and under what circumstances will dominate have remained unanswered. Conducting a set of independent trials of single-species stocks (intraspecific interactions) and mixed stocks (interspecific interactions) of marbled crayfish in combination with other four crayfish species invasive to Europe we evaluated survival, growth, claw injury, and reproduction. In both single and mixed stocks, red swamp crayfish and common yabby grew faster than marbled crayfish, while marbled crayfish were superior to both spiny-cheek and signal crayfish in terms of growth. Except for the trial with signal crayfish, the faster-growing species consistently reached a higher survival rate. Thus, the success of the marbled crayfish is significantly driven by its relatively fast growth as well as early and frequent reproduction. Our results indicate how interactions between invasive populations can unfold in the future and underline the complex population dynamics between existing and emerging invasive species.

**Abstract:**

Biological invasions are increasingly recognized ecological and economic threats to biodiversity and are projected to increase in the future. Introduced freshwater crayfish in particular are protruding invaders, exerting tremendous impacts on native biodiversity and ecosystem functioning, as exemplified by the North American spiny-cheek, signal and red swamp crayfish as well as the Australian common yabby. The marbled crayfish is among the most outstanding freshwater crayfish invaders due to its parthenogenetic reproduction combined with early maturation and high fecundity. As their introduced ranges expand, their sympatric populations become more frequent. The question of which species and under what circumstances will dominate in their introduced communities is of great interest to biodiversity conservation as it can offer valuable insights for understanding and prioritization of management efforts. In order to examine which of the aforementioned species may be more successful as an invader, we conducted a set of independent trials evaluating survival, growth, claw injury, and reproduction using single-species stocks (intraspecific interactions) and mixed stocks (interspecific interactions) of marbled crayfish vs. other crayfish invaders since the onset of exogenous feeding. In both single and mixed stocks, red swamp crayfish and yabby grew faster than marbled crayfish, while marbled crayfish were superior to both spiny-cheek and signal crayfish in terms of growth. With the exception of signal crayfish, the faster-growing species consistently reached a higher survival rate. The faster-growing species tended to negatively impair smaller counterparts by greater claw injury, delayed maturation, and reduced fecundity. Only marbled crayfish laid eggs as early as 14 weeks in this study, which is earlier than previously reported in the literature. Thus, the success of marbled crayfish among invasive crayfish is significantly driven by relatively fast growth as well as an early and frequent reproduction. These results shed light on how interactions between invasive populations can unfold when their expansion ranges overlap in the wild, thereby contributing to the knowledge base on the complex population dynamics between existing and emerging invasive species.

## 1. Introduction

The accelerating rates of international trade, travel, and transport are leading to a mixing of biota across the world and the number of species introduced to new regions continues to increase worldwide [1,2,3]. This is true for many taxonomic groups [4,5,6,7]. Some of these introduced organisms become established and invasive in new environments, continuing to spread and negatively affecting their introduced environment [8,9]. The economic costs related to such biological invasions are overwhelming and remain underestimated in many cases [10,11]. More importantly, these invasions often result in irreversible changes of newly occupied ecosystems. Thus, they are considered as one of the major threats of biodiversity and ecosystem functioning globally [12,13,14].

In the face of overwhelming numbers of introduced and invasive species [15], the need to better understand interspecific interactions among invaders in recipient ecosystems has never been greater. While many of these interactions (antagonistic, mutualistic, or competitive) have been primarily described comparing invasive species and their native counterparts, interactions among invasive species have received less attention [16]. In these complex scenarios, more opportunistic life-history strategies (‘*r-selected*’) of invasive species can facilitate their invasion success [17,18]. The study of life-history traits of emerging invasive species vs. already established invasive species is, therefore, of vital importance to predict the outcome of introductions in the future.

Crayfish (Decapoda: Astacidea) are a highly diverse taxonomic group of freshwater organisms [19] with important ecological roles in freshwater ecosystems [20,21]. Numerous crayfish species have been introduced worldwide and exerted serious negative impacts on resident biodiversity. For instance, a large number of populations of indigenous crayfish species (ICS) in Europe have been lost, and many more have been substantially reduced, due to direct or indirect effects of non-indigenous crayfish species (NICS), primarily of North American origin, such as spiny-cheek crayfish *Faxonius limosus* (Rafinesque, 1817), signal crayfish *Pacifastacus leniusculus* (Dana, 1852), and red swamp crayfish *Procambarus clarkii* (Girard, 1852) [22,23]. These species have relatively long introduction histories on the European continent, where they are widely spread [24,25], and are listed as invasive alien species of EU concern [26,27]. North American crayfish transmit the causative agent (*Aphanomyces astaci* Schikora) of the crayfish plague, a deadly disease to all crayfish not originating from North America [28]. Crayfish plague transmission aside, previous studies demonstrated the superiority of NICS over European ICS [29,30,31], in terms of higher aggressiveness and dominance in mutual interactions, higher competitiveness for resources such as food and shelter, faster growth and maturation, shorter egg incubation, and higher fecundity accompanied with a broader environmental tolerance [23,32,33].

Over the last decades, several more NICS than the three aforementioned species have been introduced in Europe. For instance, Kouba et al. [25] listed a total of seven further successfully established NICS, such as *Faxonius virilis* (Hagen, 1870), *F. juvenilis* (Hagen, 1870), and *F. immunis* (Hagen, 1870), *Cherax destructor* Clark, 1936, *C. quadricarinatus* (von Martens, 1868), *Procambarus acutus* (Girard, 1852), and *P. virginalis* Lyko, 2017, with newly introduced populations appearing frequently across the continent [34,35]. As the number of NICS and their ranges expand, novel assemblages containing multiple NICS gradually occur [34,36,37]. However, studies simultaneously comparing life-history trait differences and possible interactions among multiple NICS are particularly limited [30,38,39,40]. Among newly appearing species, the parthenogenetic marbled crayfish *P. virginalis* has become particularly widespread and concerning due to its reproduction strategy when theoretically even a single individual can establish a new population [37,41].

Using single-species and mixed stocks of marbled crayfish in combination with the spiny-cheek crayfish, the signal crayfish, the red swamp crayfish, and the Australian common yabby *C. destructor*, we conducted a set of independent laboratory experiments directly comparing their survival, growth, claw injury, and reproduction (glair gland formation, ovulation, and fecundity) since the onset of exogenous feeding. With this approach, we aim to investigate the effect of intra- vs. interspecific competition between marbled crayfish and other prominent well-established NICS on life-history traits, which may further affect the invasion success of these species. We hypothesize that the marbled crayfish as a relatively ‘new’ NICS will be able to outcompete other NICS counterparts because of its life-history strategy (parthenogenetic reproduction combined with early maturation and high fecundity). Given that these life-history traits are critical to understand how any population fares along with their potential to establish leading to a successful invasion, we considered such a comparative analysis as key in advancing our understanding of the mechanisms determining dominance of an invader in a new ecosystem.

## 2. Materials and Methods

### 2.1. Origin of Experimental Animals and Selection of Juveniles

Ovigerous females of spiny-cheek crayfish and signal crayfish were obtained from their established populations in the Czech Republic, Elbe River, Černěves (50.462° N, 14.237° E), and Stržek pond, Kozlov (49.378° N, 16.084° E), respectively, and were transported to the laboratory several weeks before hatching and gradually acclimatized to the experimental temperature. The remaining species originated from our laboratory cultures held at the Research Institute of Fish Culture and Hydrobiology, Vodňany, given their lack of wild populations in the country. The species in the laboratory culture were derived from local pet trade agents and were cultured for several generations under closed control conditions. Females of marbled crayfish and the respective counterparts were selected using those individuals with juveniles first reaching independence (i.e., those being on the onset of exogenous feeding) on the same day to ensure exact comparability of species performances in the experiment. Thus, the selection protocol did not allow the involvement of juveniles originating from females at different stages.

### 2.2. Experimental Design

**Figure 1:**
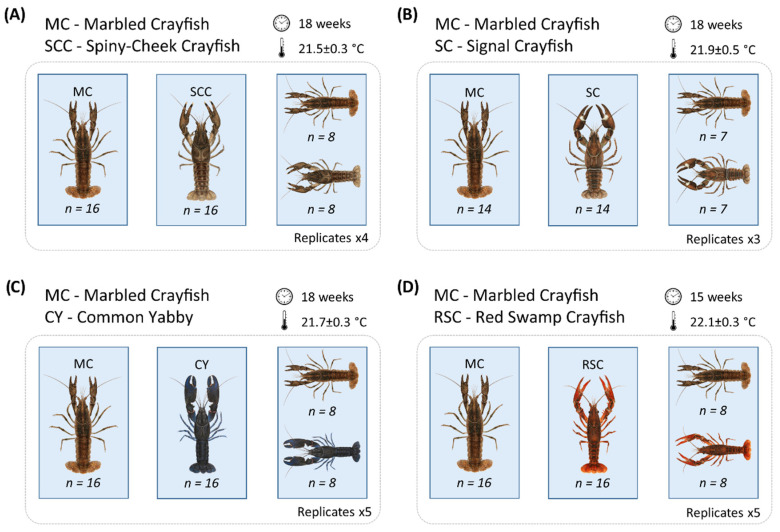
Experimental design and details of trials comparing survival, growth, claw injury, and reproduction of marbled crayfish (MC) vs. spiny-cheek crayfish (SCC, trial **A**), signal crayfish (SC, trial **B**), common yabby (CY, trial **C**), and red swamp crayfish (RSC, trial **D**) since the onset of exogenous feeding. Water temperature is presented as mean ± SD. The duration of the experiment and replicates for each trial is also shown. Note that crayfish sizes are not scaled.

In order to better understand how juveniles of different NICS perform when living in sympatry, we conducted a series of four independent pairwise laboratory experiments comparing growth, survival, claw injury, glair gland formation, ovulation, and fecundity of marbled crayfish and four prominent NICS (spiny-cheek crayfish, signal crayfish, common yabby, and red swamp crayfish) since the onset of exogenous feeding juvenile stage 2 in the signal crayfish and juvenile stage 3 in remaining species [42]. Each trial involving marbled crayfish and other NICS contained three experimental groups—two monocultures—one for each species, thereafter indicated as “single” stocks, and their communal stock with identical final stocking density and a species ratio 1:1 (thereafter indicated as “mixed” stock). The stocking density per aquarium, number of replications for all tested groups, duration of trials in weeks, and water temperature are shown Figure 1. Due to the limited number of offspring, initial stocking density and number of replications were lowered, especially in the trial involving the signal crayfish. In this trial, the initial weight of marbled crayfish juveniles is missing (we avoided initial stocking of weighed juveniles due to possible unnoticed injury prior the experiment; in other cases, we weighed a sample of 14 to 34 juveniles). All trials lasted 18 weeks, except for the trial with red swamp crayfish, which was terminated at 15 weeks due to earlier maturation of both tested species (see the Results section). The mean water temperature (registered hourly using Minikin loggers, Environmental Measuring Systems, Brno, Czech Republic) ranged from 21.5 to 22.1 °C in all trials (Figure 1). Dissolved oxygen measured daily (Oxi 315i, WTW GmbH, Weilheim, Germany), was always above 7 mg·L^−1^, usually exceeding 8 mg·L^−1^ (saturation above 90%). The daily monitored pH (pH 315i, WTW GmbH, Weilheim, Germany) was stable, ranging from 7.31 to 7.88. The light:dark regime was 14:10 h, mimicking the light regime of the growing season.

### 2.3. Culturing Conditions and Feeding

Unsexed juveniles were randomly stocked to the laboratory recirculating system with glass aquaria (37 cm width × 55 cm length × 31.5 cm height, the usable volume of 55 L) representing culture units (experimental details summarized in the Figure 1). To minimize aggression and cannibalism, shelters were provided by one fired clay brick (6.5 × 28.5 × 13.5 cm) with 39 cross holes (26 and 13 holes with a profile of 1 × 3 cm and 1 × 1 cm, respectively) placed in each aquarium [43], i.e., the number of available individual shelters exceeded stocked juveniles more than twice at the beginning of the experiment. As the crayfish grew during the experiment, two blocks of joined polypropylene tubes, each containing five tubes (length 10 cm, inner diameter 35 mm), were added as larger shelters at six weeks of culture. The base of each block was represented by three longitudinally-joined tubes with two further tubes positioned pyramidal in the second layer [44].

In all trials, crayfish were fed ad libitum daily by defrosted chironomid larvae and pond zooplankton for the first six weeks. Fresh plankton was obtained from a local pond when the particular trial started and kept frozen at −20 °C until utilization (see Table S1 for plankton species composition). After six weeks, this diet was altered to defrosted chironomid larvae and commercial pellets (Granugreen, Sera, Heinsberg, Germany). Aquaria were cleaned three times a week (Monday, Tuesday, and Friday). To minimize handling with the animals, individual dry weights (removal of excessive water on the absorbing tissue paper) were weighed every three weeks by using analytical balance (Kern & Sohn GmbH, Balingen, Germany) to the nearest 0.001 g. On this occasion, animals were also checked for missing and regenerating claws. Once glair glands were noticed, the reproduction status of females was assessed weekly (presence of glair glands and number of eggs after detachment from pleopods by tweezers—both absolute and relative fecundities [per mm of carapace length] were analyzed).

### 2.4. Statistical Analyses

All data were assessed for normality and homoscedasticity using Kolmogorov–Smirnov and Levene’s tests, respectively. Given the lack of test prerequisites (normality data and/or homoscedasticity in several groups), weights in single and mixed stocks were compared with non-parametric Kruskal–Wallis tests followed by multiple comparisons of mean ranks for all groups as a post-hoc test. Pairwise intraspecific comparisons (single vs. mixed stocks) in given trials and time were also evaluated using the non-parametric Mann–Whitney U test. Sex-intraspecific weight differences at the end of the trials were compared using Student’s t-test in sexually reproducing species. The absolute and relative fecundity in single vs. mixed stocks of marbled crayfish were compared using Student’s t-test as well. These analyses were performed in Statistica software 12.0 for Windows (StatSoft, Prague, Czech Republic). We used a non-parametric survival analysis (Kaplan–Meier method) using the ‘*survival*’ R package [45] and tested for significant differences between specific pair assemblages. In addition, to assess the incidence of individuals with missing and/or regenerating claws (those individuals indicated as 1; intact animals considered as 0), we run generalized linear models with quasibinomial distribution due to overdispersion of data [46] followed by post-hoc tests to determine possible differences in the given assemblages through time and between groups (single and mixed stocks of tested species in the given time). For all statistical tests, *p*-values < 0.05 were considered significant.

## 3. Results

### 3.1. Growth Analysis

The initial weight of the stocked juveniles at the onset of exogenous feeding varied greatly among species. At the juvenile stage 3, the individual weight of marbled crayfish generally ranged between 5 and 6 mg, while spiny-cheek crayfish and red swamp crayfish were nearly twice as big (9.8 ± 0.5 and 9.9 ± 1.2 mg, respectively) and common yabby three times as big (15.2 ± 1.0 mg). Although initial weight for marbled crayfish was not available in the trial with signal crayfish, based on the other trials, stocked signal crayfish at juvenile stage 2 would have been around four times bigger (21.3 ± 1.7 mg) than marbled crayfish at juvenile stage 3 (see Figure 1 for details).

**Figure 2:**
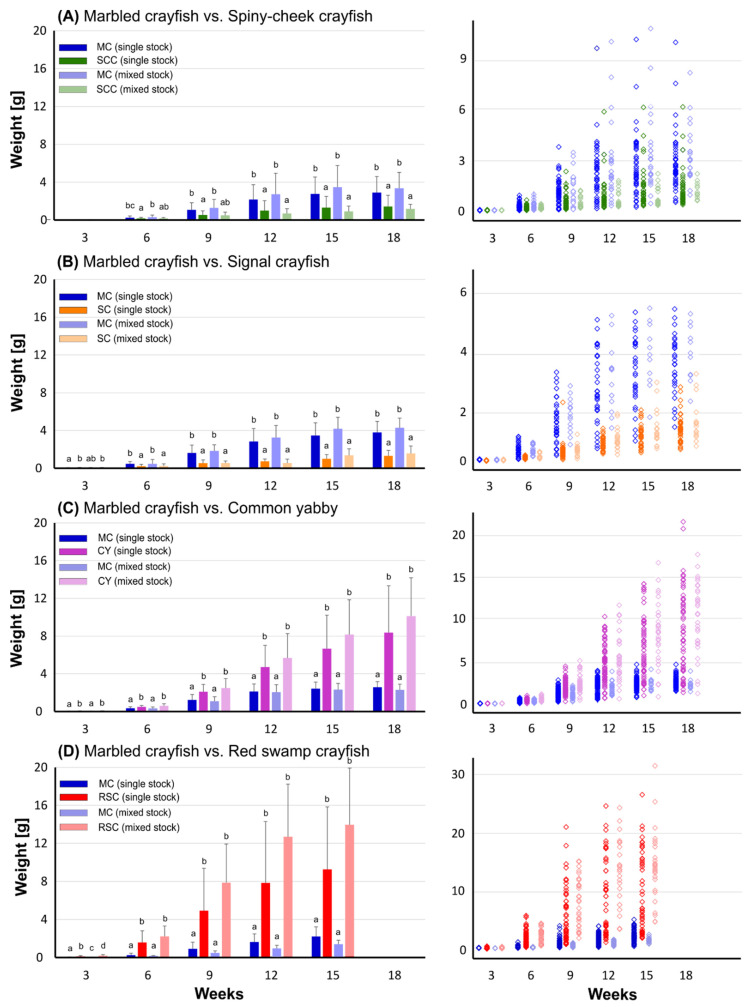
Weight (g) of marbled crayfish—MC in trials with spiny-cheek crayfish—SCC (**A**), signal crayfish—SC (**B**), common yabby—CY (**C**), and red swamp crayfish—RSC (**D**) kept in single-species and mixed stocks through the experiment (note that darker and lighter colors are used for single and mixed stocks, respectively). The left side of the figure panel represents the mean values (+SD). Where applicable, differing letter superscripts (a, ab, b, bc, c, d) indicate statistical differences (Kruskal–Wallis test, Multiple comparisons of mean ranks for all groups, *p* < 0.05) among groups in the given time. Note the uniform scale of the Y-axis for better comparability of trials. The right side of the figure panel shows individual values with adjusted scales to better depict the intraspecific variabilities observed.

Despite the apparent initial disadvantage of marbled crayfish in terms of size, it grew consistently faster than signal crayfish as well as spiny-cheek crayfish but not when compared to common yabby and red swamp crayfish (Figure 2). Except for the beginning of the trial with the spiny-cheek crayfish (week 3), significant interspecific differences in weight were always apparent. In general, species with faster growth tended to attain greater sizes in the mixed stocks when contrasted with their monocultures and vice versa, as indicated by the mean as well as individual weight data (Figure 2), but these differences were not usually significant.

Final maximum values of weight rarely exceeded 5 g in the marbled crayfish across all trials and were suppressed by the onset of female maturation and reproduction (see results below). Yet, individuals up to 10 g were noticed in the trial with spiny-cheek crayfish (the smallest species on average), whose individual weights varied largely in the single stock but not in the mixed stock. Signal crayfish, as well as marbled crayfish in the trials with common yabby and red swamp crayfish, respectively, were also relatively consistent in weight. At the experiments, common yabby and red swamp crayfish even exceeded 20 and 30 g after 18 and 15 weeks, respectively (Figure 2).

**Figure 3:**
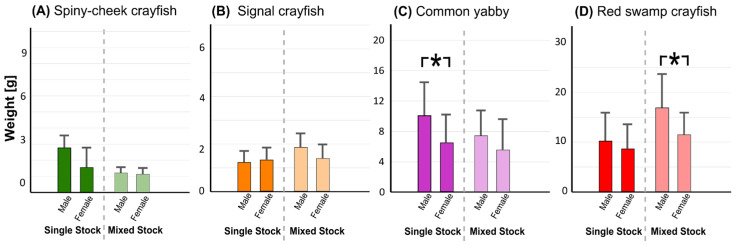
Sex-related final weight in sexually reproducing species (spiny-cheek crayfish (trial **A**), signal crayfish (**B**), common yabby (**C**), and red swamp crayfish (**D**)) kept in single-species and mixed stocks. Asterisks (*) indicate significant difference between sexes in the given stock, Student’s *t*-test, *p* < 0.05.

When considering sex-related weight differences in individuals surviving until the end (with the exception of parthenogenetic marbled crayfish), the mean absolute values of males were on average higher in all but one group—signal crayfish kept in single stock (Figure 3). However, significant differences were achieved only in the single stock of common yabby and mixed stock of red swamp crayfish (Figure 3).

### 3.2. Survival Rate

**Figure 4:**
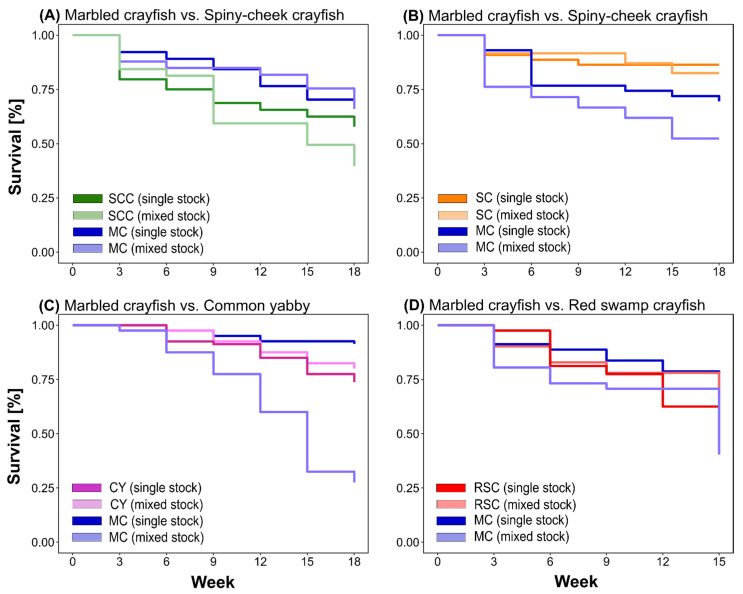
Kaplan–Meier survival analysis plots of marbled crayfish (MC) in trials with spiny-cheek crayfish (SCC) (**A**), signal crayfish—SC (**B**), common yabby—CY (**C**), and red swamp crayfish—RSC (**D**) kept in single-species and mixed stocks through the experiment (note that darker and lighter colors are used for single and mixed stocks, respectively).

The final survival rates of marbled crayfish kept as single stocks ranged from 69% (trials with signal crayfish and spiny-cheek crayfish) to 91% (trial with common yabby). The lowest final survival was observed in the single stocks of spiny-cheek crayfish (59%), followed by red swamp crayfish (60%), while higher final survival was observed in the common yabby (74%) and signal crayfish (83%; see Figure S1). As derived from survival analyses modeling, we identified significant differences between the survival of NICS among all assemblages (Figure 4, for statistical values Table S2). Marbled crayfish generally expressed a higher survival rate over the experiment than the spiny-cheek crayfish, but had a lower survival rate compared to signal crayfish in mixed stocks. We found no significant differences between the survival rate of either of these NICS when grown in single stocks, nor between single and mixed stocks of either species. However, marbled crayfish expressed lower survival rates compared to the common yabby and red swamp crayfish when housed in mixed but not in single stocks. When in a mixed stock with common yabby, the marbled crayfish showed a significantly lower survival rate compared to its survival in single stock. Red swamp crayfish in the mixed stock expressed a significantly higher survival rate compared to its single stock (Figure 4, Table S2).

### 3.3. Missing and/or Regenerating Claws

In all but one case (species assemblage in trial A with the spiny-cheek crayfish), the model outputs indicated significant differences in species assemblages, time, and their interactions in terms of claw injuries (Table S3). Overall, the incidence of claw injuries increased over time, with the exception of spiny-cheek crayfish in the single stock, and marbled crayfish in the mixed stock (trail with signal crayfish) as well as single stock (trial with common yabby) (see Table 1). Incidence of missing and/or regenerating claws did not exceed 20% in survivors within the single stocks of marbled crayfish, spiny-cheek crayfish, and signal crayfish, but was substantial in rapidly growing species with greater individual weight variance, resulting in up to 37% and 42% missing and/or regenerating claws in the common yabby and red swamp crayfish, respectively (Table 1). Marbled crayfish were less damaged in the mixed stocks with the signal crayfish and vice versa at weeks 15 and 18. More balanced situation occurred in the trial with spiny-cheek crayfish. On the other hand, marbled crayfish suffered up to 45% and 50% incidence in mixed stocks with common yabby and red swamp crayfish at the end of the trials, respectively, which in turn led to substantial but significantly fewer claw injuries (up to 22% and 35%, respectively) compared to their single stocks (Table 1).

### 3.4. Speed of Maturation and Fecundity Rates

Development of glair glands was first noticed in the marbled crayfish (week 11; an accidental finding out of regular weighing scheme) followed by red swamp crayfish (week 12), spiny-cheek crayfish (week 13), and common yabby (week 15). No glair glands were found in signal crayfish (Table 2). In general, glair gland formation followed a pattern of earlier onset and/or more rapid development when the species was superior in weight, as in the case of marbled crayfish mixed stocks in trials with the spiny-cheek crayfish and signal crayfish, or common yabby and red swamp crayfish when kept with marbled crayfish. In these cases, animals tended to perform better than their respective monocultures (considering that observed values in mixed stocks are derived from lower absolute numbers of stocked animals). These patterns were also pronounced in reproduction performance of marbled crayfish, the only species which laid eggs in our experiment. While not statistically significant, its absolute and relative pleopodal fecundities tended to be higher when cultured together with signal crayfish (increased by 25% and 16%, respectively) and spiny-cheek crayfish (12% and 9%, respectively) (Table 2). Only a slight reduction (decline by 4 and 5%, respectively) was observed when kept together with the common yabby. Additionally, no marbled crayfish laid eggs when kept together with the superior red swamp crayfish (Table 2). Red swamp crayfish mated regularly at the end of the experiment (week 15).

## 4. Discussion

Growth and survival rates, speed of maturation, mode of reproduction, and fecundity are among the principal life history traits determining the success of any species [31,47,48]. These attributes are often of special interest when indigenous and non-indigenous species interact, or when more non-indigenous (and potentially invasive) species co-occur, possibly resulting in the disappearance of one species. For instance, several populations of European ICS have been lost, and many more have been substantially reduced, largely due to the direct or indirect effects of NICS [22,23]. This is because introduced freshwater crayfish often exhibit invasive behavior in their non-native ranges [49]. As the number of NICS gradually increases in the European continent, and their ranges expand, new sympatric populations steadily appear [34,40,50]. Unlike between native and invasive crayfish species, the interactions between NICS when these co-occur are not yet well understood.

### 4.1. Intra- and Interspecific Growth and Survival Rates in Single and Mixed Stocks

Despite being the smallest at the onset of exogenous feeding generally between 5 to 6 mg [51], marbled crayfish was capable to outgrow spiny-cheek crayfish and signal crayfish but not common yabby and especially not red swamp crayfish, which grew substantially faster than marbled crayfish. While usually not significantly different, faster-growing species tended to attain greater sizes in the mixed stocks when compared to their monocultures and vice versa. Our results suggest substantial interspecific differences in the growth rates, but also a role of different intraspecific stocking densities of the species—greater sizes of the faster growing species (e.g., common yabby and red swamp crayfish) can be more easily achieved in the mixed stock with a smaller counterpart e.g., marbled crayfish (and thus lowered intraspecific interactions) when compared with their monoculture. Males tended to attain greater weight than females, as expected in crayfish species [52].

The marbled crayfish is a middle-sized species with high growth potential under favorable conditions. Its laboratory stocks as well as wild populations usually do not exceed 10 cm of total body length (~20 g). Larger size classes (e.g., up to 12–13 cm) can be found in wild populations [50,53], but the abundance of such size classes is typically low [54,55,56,57]. In our study, final weight rarely exceeded 5 g (~6 cm total body length) with the greatest values of 10 g, corresponding to ~7.5 cm total body length [58], achieved in the trial with the spiny-cheek crayfish. The growth rate of marbled crayfish was hampered by its early maturation and frequent reproduction with the onset of glair gland formation commonly seen since week 12 in our experiment. The spiny-cheek crayfish was on average the smallest species in our trials when compared to the marbled crayfish, where individual weight varied largely in the single stock but not the mixed stock, presumably suppressed by the presence of larger marbled crayfish. Similar to the marbled crayfish, the spiny-cheek crayfish is also a middle-sized species that typically does not exceed 9 to 10 cm [52], although individuals with a body length reaching 13 cm have also been reported [59,60]. A small portion of fast-growing individuals can achieve 4 to 5 cm at the end of their first growing season [31]. This concurs with our results, with the largest animal weighing more than 6 g, corresponding to ~6 cm of total body length. The signal crayfish is a rather large crayfish species with females and males measuring up to 12 cm and 16 cm, respectively. Yet, the upper weight limit can be considerable, ranging from 200 to 250 g [61,62,63]. Westman et al. [64] reported the mean size of juveniles at the end of their first season to reach 3 cm, noting though that it may differ among populations. For example, Abrahamsson [65] reported a size of 4 cm for one-year-old crayfish. This is concomitant with our data, where several individuals exceeded a weight of 2.5 g (>4 cm).

The common yabby is a relatively large species, with a total body length usually not exceeding 15 cm and a weight of 150 g. However, individuals weighing up to 350 g can rarely be observed [61]. In our study, the species successfully exceeded 20 g in 18 weeks; thus, it is not surprising that this warm-water species is of great interest to crayfish aquaculture [66,67]. However, global crayfish aquaculture is mostly focused on the red swamp crayfish [68,69]. Its total body length usually ranges from 10 to 12 cm, but can reach up to 20 cm in exceptional cases [61]. Its growing potential is enormous compared to the aforementioned species, almost doubling its weight on average every three weeks when kept in single stocks, or even quadrupling its weight from week 6 to 9 in mixed stocks. In fact, juveniles can reach 50 g in just three to five months [23], which coincides with our findings of one animal exceeding 30 g within just 15 weeks. Laboratory experiments indicated similar behavioral competencies of both species in interactions when similar-sized individuals were compared. However, the red swamp crayfish seems superior, primarily owing to its greater growth rates and size achieved at adulthood. Recent research suggests a superior position of the red swamp crayfish at sympatric localities [34] as well as enhanced predation on the marbled crayfish [40].

Despite the standardization among conducted trials, these should be primarily considered as pairwise comparisons with the marbled crayfish, given that this species performed differently in the evaluated indices through trials. For instance, the composition of the plankton provided varied, so its replacement with a more standardized fresh diet is advised in future studies for better comparability (e.g., *Artemia* sp. nauplii). A key factor in the growth of crustaceans, and crayfish in particular, is temperature. An increase of temperature normally accelerates growth by shortening the inter-molt period and raising the frequency of molting [70,71]. Despite keeping the temperature constant in our experiments, different intra- and interspecific growth patterns were identified. It is well demonstrated that growth and life-history traits of those warm-water crayfish species (i.e., common yabby and red swamp crayfish) are more likely to push the limits of *r*-type strategies, while those from colder waters (i.e., spiny-check and signal crayfish) do so to a lesser extent [72]. Our results not only support the dominance of marbled crayfish over rather cold-water species when cohabiting, and vice versa over typically warm-water species, but also the large intraspecific variability in growth rate.

Survival rates developed in close relation with the growth performance discussed earlier. The faster-growing species usually tended to reach a higher survival rate in the mixed stocks when compared with their monocultures (because of lower intraspecific density in the defined culturing conditions), while survival of the smaller counterpart was negatively impaired. An interesting situation developed in the trial with signal crayfish, which, regardless of its smaller size, retained a very high survival rate. This can be explained by the presumed space segregation when signal crayfish occupied smaller shelters in bricks which larger marbled crayfish could not enter effectively. It can be presumed that a shortage in critical resources such as shelters and food would result in the reduced survival of signal crayfish as well.

### 4.2. Incidence of Missing and/or Regenerating Claws

Adding to the context of growth and survival rates, it is important to discuss the incidence of missing and/or regenerating claws as a consequence of individual interactions. Aggressiveness has often been associated with the success of invasive species, as well as with enhancing their competitiveness in a novel environment, hence helping them expand in their invaded range [73,74]. In our case, the incidence of missing and/or regenerating claws was relatively low in single stocks of marbled crayfish, spiny-cheek crayfish, and signal crayfish, but it was substantial in fast-growing species with a broad individual weight variability. Importantly, the presence of superior species in terms of growth in the mixed stock resulted in a greater injury rate of smaller counterparts and vice versa. These results are partially supported by previous studies, which showed that, in general, the marbled crayfish is less aggressive than other invasive species, such as the signal crayfish or the common yabby [75], but it may compete with the red swamp crayfish in equal-sized pairings [76,77]. We consider the mere weight contribution of missing and/or regeneration claws to the total weight presented, of secondary importance in the context of this study, and instead highlight the importance of the magnitude of different interspecific growth rates in the evaluated species. Notably, claws are important for mating, defense against predators, intra- and interspecific interactions, capture and manipulation of prey, burrowing, and carrying sensory structures in males that aid in the discrimination and localization of the female scent. Once injured, an animal is at greater risk of further damage and its fitness is reduced [78]. While demanding, regeneration of body appendages is well developed in crayfish [79], largely accompanied with molting. However, each molting needs to be weighed with an increased risk of natural mortality, predation, and cannibalism during this vulnerable period (note the high survival of slow-growing signal crayfish discussed earlier, i.e., lowered molting-related mortality).

### 4.3. Speed of Maturation and Fecundity Rates

Freshwater crayfish develop glair glands on the underside of the pleon prior to spawning, producing a mucus that, among others, aids in fertilization and attachment of the eggs to the pleopods [80]. The presence of glair glands was first observed in the single stock of the marbled crayfish species in the trial with common yabby at week 11 (the regular methodological time frame presumed control at week 12). The number of animals with developed glair glands (eight females) suggests that they might first appear even earlier in the species. A similar situation is also possible for the red swamp crayfish at week 12 with 11 and 3 females carrying glair glands in the single and mixed stock, respectively.

The marbled crayfish is the only species in our experiment that ovulated eggs, first seen in its monoculture when compared with the red swamp crayfish at week 14 (98 days) and from week 16 onwards in mixed stocks. If we presume that stages 1 and 2 lasted 12 days in our culturing conditions [81], it corresponds to ovulation at 110 days and possibly earlier, considering the frequency of controls. This is a month earlier than previously reported for this species—Seitz et al. [82] referred to females first reproducing at 141 to 255 days (30 weeks on average) when kept at 20–25 °C. Similar to survival and incidence of missing and/or regenerating claws, onset and dynamics of glair gland formation (as well as first ovulation and fecundity in the marbled crayfish) were related with the weight of assessed species in the mixed stocks. The marbled crayfish was most suppressed when cultured together with the red swamp crayfish, where only one female with glair glands was observed (and did not manage to ovulate eggs). Both marbled crayfish and red swamp crayfish are highly fecund species. Marbled crayfish usually carry 50 to 200 eggs, while larger females can have up to 400 eggs [58,83]. In the wild though, it is possible to find females carrying around 700 eggs [53,56,84]. Fecundity of red swamp crayfish typically ranges from 200 to 300 eggs but can reach up to 700 eggs [85,86,87].

Based on body proportions and formation of copulatory stylets (gonopods) in males, form I males and females of spiny-cheek crayfish, i.e., those that are sexually active [88,89], were first noticed at week 12. The first female with glair glands appeared a week later. A part of spiny-cheek crayfish individuals in populations can mature at the end of their first growing season, exhibiting presence of glair glands as well as mating behavior. Egg laying occurs in the next spring [31] with a commonly observed fecundity below 250 eggs, but potentially exceeding 500 eggs in exceptional cases [59,90,91]. In contrast, young-of-the-year marbled crayfish can theoretically reproduce in late summer/early autumn in the wild. The proportion of such females and the performance of this new generation during winter in temperate climates is unknown [53,92]. Signal crayfish generally reaches maturity in their second to third year at sizes ranging from 6 to 9 cm [63] and males typically mature one year earlier than females [65,93,94]. Its fecundity ranges between 100 to 400 eggs, but some females can have more than 500 eggs [94,95]. In this context, achieving maturation is comparatively a considerable disadvantage of this species compared to the other species examined in this study. However, it is substantially larger in adulthood and prefers colder localities. Competitiveness of marbled crayfish under such circumstances is worth further investigation.

Common yabby matures at a weight of about 20 g (~9 cm) and an age of less than one year. Under suitable conditions (water temperature of at least 18 to 20 °C and a photoperiod over 14 h), it is able to reproduce up to five times per year [32,96,97], usually carrying 300 to 500 eggs [98,99], but can reach up to 1400 eggs in large females [66]. We observed formation of glair glands even in smaller females (<8 cm) since week 15. While mortality might be a contributing factor, the number of females with glairs glands stayed stable in the mixed stock and declined in the single culture later on. This suggests that maturity can be achieved very rapidly, but our culturing conditions did not support reproduction of the species in our experiment. The red swamp crayfish is a typical *r*-strategist reaching maturity at an age of 4 to 5 months and a body length of less than 4.5 cm [32,100]. This species is also highly fecund and two generations per year can emerge [85,101]. We observed females with glair glands as early as week 12. At the termination of the experiment (week 15), mating behavior was common, but did not result in oviposition. Similar to the formation of glair glands in the common yabby above, it seems that the provided conditions were not suitable for completion of reproduction of these species. Yet, speed of maturation and fecundity of the red swamp crayfish was found to be very similar to that of marbled crayfish.

## 5. Conclusions

The growth rate, interaction dynamics and, in general, the life history traits of introduced species play a key role in their invasion success. Nowadays, with the increasing number of introductions, the overlap of several invasive populations is becoming more and more frequent; however, the effects of this overlap remain poorly understood. Our results show that, while greater growth of the parthenogenetic marbled crayfish occurs when it is present together with rather cold-water invasive species (signal crayfish and spiny-cheek crayfish), it is suppressed when co-occurring with typically warm-water invasive species, such as the common yabby and red swamp crayfish. Similarly, although the single stock survival rate of the marbled crayfish is generally high, its survival rate decreased sharply when cultured with fast-growing warm-water species. These results could be directly related to the incidence of claw loss and regeneration. Furthermore, despite its smaller size, the marbled crayfish reaches maturation earlier, which may represent a trade-off between growth and maturation speed in this species. When specifically contrasted with the signal crayfish, early maturation and high fecundity benefit the marbled crayfish to a great extent; however, a role of signal crayfish size at adulthood will certainly be important in their interspecific interactions. Additionally, studying the described relationships at lower temperatures representing colder localities is of great interest, as these might suit better the requirements of both spiny-cheek crayfish and signal crayfish. These experimental studies would emphasize the importance of understanding how invasive species cope with their invasive interspecific when their populations overlap under different environmental conditions. Such insights could have important implications for predicting the spread of invasive populations as well as better understanding the outcome of overlaps that may occur in the future when new emerging invasive species are introduced, thus prioritizing management efforts.

## Figures and Tables

**Table 1:** Incidence (%) of individuals with missing and/or regenerating claws of marbled crayfish—MC in trials with spiny-cheek crayfish—SCC (A), signal crayfish—SC (B), common yabby—CY (C), and red swamp crayfish—RSC (D) kept in single-species and mixed stocks through the experiment.

Trial	Group	Week					
		3	6	9	12	15	18
A	MC single	0 ^A,a^	0 ^A,a^	2 ^B,a^	20 ^C,a^	11 ^BC,a^	9 ^BC,a^
	SCC single	4 ^A,a^	6 ^A,a^	7 ^A,a^	2 ^A,a^	5 ^A,a^	11 ^A,a^
	MC mixed	0 ^A,a^	0 ^A,a^	4 ^B,a^	8 ^B,a^	8 ^B,a^	10 ^B,a^
	SCC mixed	0 ^A,a^	4 ^B,a^	11 ^B,a^	5 ^B,a^	20 ^B,a^	8 ^B,a^
B	MC single	3 ^B,a^	0 ^A,a^	16 ^B,a^	6 ^B,a^	3 ^B,b^	14 ^B,bc^
	SC single	0 ^A,a^	3 ^B,a^	3 ^B,a^	3 ^B,a^	3 ^B,b^	3 ^B,b^
	MC mixed	0 ^A,a^	0 ^A,a^	0 ^A,a^	0 ^A,a^	0 ^A,a^	0 ^A,a^
	SC mixed	0 ^A,a^	0 ^A,a^	0 ^A,a^	11 ^B,a^	35 ^BC,c^	53 ^C,c^
C	MC single	1 ^A,a^	1 ^A,a^	7 ^A,a^	5 ^A,a^	5 ^A,a^	4 ^A,a^
	CY single	0 ^A,a^	4 ^B,a^	8 ^BC,a^	13 ^BC,a^	24 ^CD,b^	37 ^D,b^
	MC mixed	0 ^A,a^	3 ^B,a^	2 ^B,a^	42 ^C,b^	23 ^BC,ab^	45 ^C,b^
	CY mixed	0 ^A,a^	10 ^B,a^	5 ^B,a^	14 ^B,ab^	16 ^B,ab^	22 ^B,ab^
D	MC single	0 ^A,a^	3 ^B,a^	6 ^B,a^	2 ^B,a^	3 ^B,a^	NA
	RSC single	0 ^A,a^	8 ^B,a^	23 ^BC,a^	32 ^C,b^	42 ^C,b^	NA
	MC mixed	3 ^A,a^	3 ^AB,a^	18 ^AB,a^	13 ^AB,ab^	50 ^B,b^	NA
	RSC mixed	0 ^A,a^	0 ^A,a^	13 ^B,a^	14 ^B,ab^	35 ^C,b^	NA

Upper indices denote differences in the given assemblages over time (uppercase letters) and between groups—single and mixed stocks of species analyzed—in the given time (lowercase letters), using GLM with quasi-binomial distribution followed by post-hoc tests, *p* < 0.05.

**Table 2:** Number of females with developed glair glands and with eggs throughout the experiment (note that absolute numbers of evaluated individuals vary; additionally, females with detached eggs miss glair glands in the following time period). Marbled crayfish (MC) in trials with spiny-cheek crayfish—SCC (A), signal crayfish—SC (B), common yabby—CY (C), and red swamp crayfish—RSC (D) kept in single-species and mixed stocks.

Trial	Group	Week								Fecundity	
		11	12	13	14	15	16	17	18	Absolute	Relative
A	MC single	–	–	3 + 0	13 + 0	15 + 0	15 + 8	17 + 3	10 + 12	134 ± 66	5.5 ± 2.0
	SCC single	–	–	1 + 0	2 + 0	3 + 0	3 + 0	3 + 0	3 + 0	–	–
	MC mixed	–	1 + 0	1 + 0	9 + 0	9 + 0	11 + 2	10 + 2	8 + 5	150 ± 65	6.0 ± 1.9
	SCC mixed	–	–	–	–	1 + 0	1 + 0	1 + 0	1 + 0	–	–
B	MC single	–	–	–	–	5 + 0	11 + 1	16 + 2	12 + 6	220 ± 82	8.6 ± 2.8
	SC single	–	–	–	–	–	–	–	–	–	–
	MC mixed	–	–	–	–	3 + 0	7 + 0	4 + 0	6 + 4	275 ± 50	10.0 ± 1.6
	SC mixed	–	–	–	–	–	–	–	–	–	–
C	MC single	8 + 0	18 + 0	21 + 0	39 + 0	29 + 19	24 + 17	14 + 17	11 + 2	131 ± 42	6.1 ± 1.8
	CY single	–	–	–	–	5 + 0	16 + 0	14 + 0	8 + 0	–	–
	MC mixed	–	1 + 0	6 + 0	8 + 0	8 + 0	7 + 5	5 + 2	2 + 2	126 ± 39	5.8 ± 1.4
	CY mixed	–	–	–	–	4 + 0	9 + 0	9 + 0	9 + 0	–	–
D	MC single	–	11 + 0	17 + 0	17 + 1	9 + 8	NA	NA	NA	126 ± 32	6.1 ± 1.0
	RSC single	–	1 + 0	3 + 0	5 + 0	3 + 0	NA	NA	NA	–	–
	MC mixed	–	1 + 0	1 + 0	0 + 0	0 + 0	NA	NA	NA	–	–
	RSC mixed	–	3 + 0	6 + 0	8 + 0	8 + 0	NA	NA	NA	–	–

The last columns indicate absolute and relative (per mm of carapace length) pleopodal fecundity presented as mean ± SD. Fecundity did not differ significantly between single and mixed stocks of marbled crayfish in evaluated indices (Student’s *t*-test, *p* < 0.05).

## Data Availability

The data that support the findings of this study are available from the corresponding author upon reasonable request.

## Supplementary Materials

The following are available online at https://www.mdpi.com/article/10.3390/biology10050422/s1, Table S1. The estimated share of abundances of planktonic organisms provided to crayfish compared to marbled crayfish during the first six weeks of experimental trials, Table S2. The output of the Kaplan-Meier survival analysis of marbled crayfish—MC in trials with spiny-cheek crayfish—SCC (A), signal crayfish—SC (B), common yabby—CY (C), and red swamp crayfish—RSC (D) kept in single-species and mixed stocks through the experiment, Table S3. The output of the GLM with quasibinomial distribution on the incidence of individuals with missing and/or regenerating claws of marbled crayfish—in trials with spiny-cheek crayfish—A, signal crayfish—B, common yabby—C, and red swamp crayfish—D kept in tested assemblages through the experiment, Figure S1. Survival (%) of marbled crayfish—MC in trials with spiny-cheek crayfish—SCC (A), signal crayfish—SC (B), common yabby—CY (C), and red swamp crayfish—RSC (D) kept in single-species and mixed stocks through the experiment (note darker and lighter colors used for single and mixed stocks, respectively).
