# Supplementary material for: Survival, Growth, and Reproduction: Comparison of Marbled Crayfish with Four Prominent Crayfish Invaders
Source: Biology (Basel). 2021 May 10;10(5):422. doi: 10.3390/biology10050422 (PMC8151088; doi:10.3390/biology10050422)
Supplement: Supplementary file 1 [file biology-10-00422-s001.zip › biology-1186474-supplementary.pdf]

Supplementary Materials

Survival, Growth, and Reproduction: Comparison of Marbled Crayfish with Four Prominent Crayfish Invaders

Antonín Kouba [1,*], Boris Lipták [1], Jan Kubec [1], Martin Bláha [1], Lukáš Veselý [1], Phillip J. Haubrock [1,2], Francisco J. Oficialdegui [3], Hamid Niksirat [1], Jiří Patoka [4] and Miloš Buřič [1]

[1] South Bohemian Research Center of Aquaculture and Biodiversity of Hydrocenoses, Faculty of Fisheries and Protection of Waters, University of South Bohemia in České Budějovice, Zátiší 728/II, 38925 Vodňany, Czech Republic; liptaq.b@gmail.com (B.L.); kubecj@frov.jcu.cz (J.K.); blaha@frov.jcu.cz (M. Bláha); veselyl@frov.jcu.cz (L.V.); phillip.haubrock@senckenberg.de (P.J.H.); niksirat@frov.jcu.cz (H.N.); buric@frov.jcu.cz (M. Buřič)

[2] Department of River Ecology and Conservation, Senckenberg Research Institute and Natural History Museum Frankfurt, Clamecystrasse 12, 63571 Gelnhausen, Germany

[3] Department of Wetland Ecology, Doñana Biological Station (EBD-CSIC), C/Américo Vespucio 26, Isla de la Cartuja, 41092 Seville, Spain; oficialdegui@ebd.csic.es

[4] Department of Zoology and Fisheries, Faculty of Agrobiology, Food and Natural Resources, Czech University of Life Sciences Prague, Kamýcká 129, 16500 Prague-Suchdol, Czech Republic; patoka@af.czu.cz

* Correspondence: akouba@frov.jcu.cz

Table S1. The estimated share of abundances of planktonic organisms provided to crayfish compared to marbled crayfish during the first six weeks of experimental trials.

Compared crayfish	Organism	Proportion (%)
Spiny-cheek crayfish	*Daphnia magna*	30
	D. pulicaria	25
	D. galeata	10
	Ceriodaphnia sp.	10
	Eudiaptomus sp.	10
	Volvox sp.	10
	Scapholeberis mucronata	5
Signal crayfish	*Bosmina longirostris*	50
&	*Cyclops vicinus* (adults and copepodits)	49
Red swamp crayfish	*Chydorus ovalis*, *Alona* sp.	< 1
Common yabby	*B. longirostris*	98
	Acanthocyclops americanus	1
	Daphnia longispina	< 1

Table S2. The output of the Kaplan-Meier survival analysis of marbled crayfish – MC in trials with spiny-cheek crayfish – SCC (A), signal crayfish – SC (B), common yabby – CY (C), and red swamp crayfish – RSC (D) kept in single-species and mixed stocks through the experiment.

Trial	Test	Chisq	df	p
A	Overall	8.1	3	0.04
	MC single vs SCC single	2	1	0.2
	MC mixed vs SCC mixed	4.5	1	0.03
	MC single MC mixed	0	1	0.8
	SCC single vs SCC mixed	1.8	1	0.2
B	Overall	10.3	3	0.02
	MC single vs SC single	3.1	1	0.08
	MC mixed vs SC mixed	4.7	1	0.03
	MC single MC mixed	2.1	1	0.2
	SC single vs SC mixed	0.1	1	0.7
C	Overall	63.8	3	<0.001
	MC single vs CY single	8.2	1	0.004
	MC mixed vs CY mixed	21.4	1	<0.001
	MC single MC mixed	55.5	1	<0.001
	CY single vs CY mixed	0.5	1	0.5
D	Overall	14	3	0.003
	MC single vs RSC single	4.2	1	0.04
	MC mixed vs RSC mixed	4.2	1	0.04
	MC single MC mixed	0.2	1	0.7
	RSC single vs RSC mixed	13.8	1	<0.001

Table S3. The output of the GLM with quasibinomial distribution on the incidence of individuals with missing and/or regenerating claws of marbled crayfish – in trials with spiny-cheek crayfish – A, signal crayfish – B, common yabby – C, and red swamp crayfish – D kept in tested assemblages through the experiment.

Trial	Test	F	df	p
A	Time	5.560	5	< 0.001
	Species assemblage	0.506	3	0.678
	Time × Species assemblage	2.012	15	0.012
B	Time	8.978	5	< 0.001
	Species assemblage	17.002	3	< 0.001
	Time × Species assemblage	2.216	15	0.005
C	Time	21.740	5	< 0.001
	Species assemblage	16.684	3	< 0.001
	Time × Species assemblage	1.869	15	0.022
D	Time	24.342	5	< 0.001
	Species assemblage	23.464	3	< 0.001
	Time × Species assemblage	2.098	12	0.015

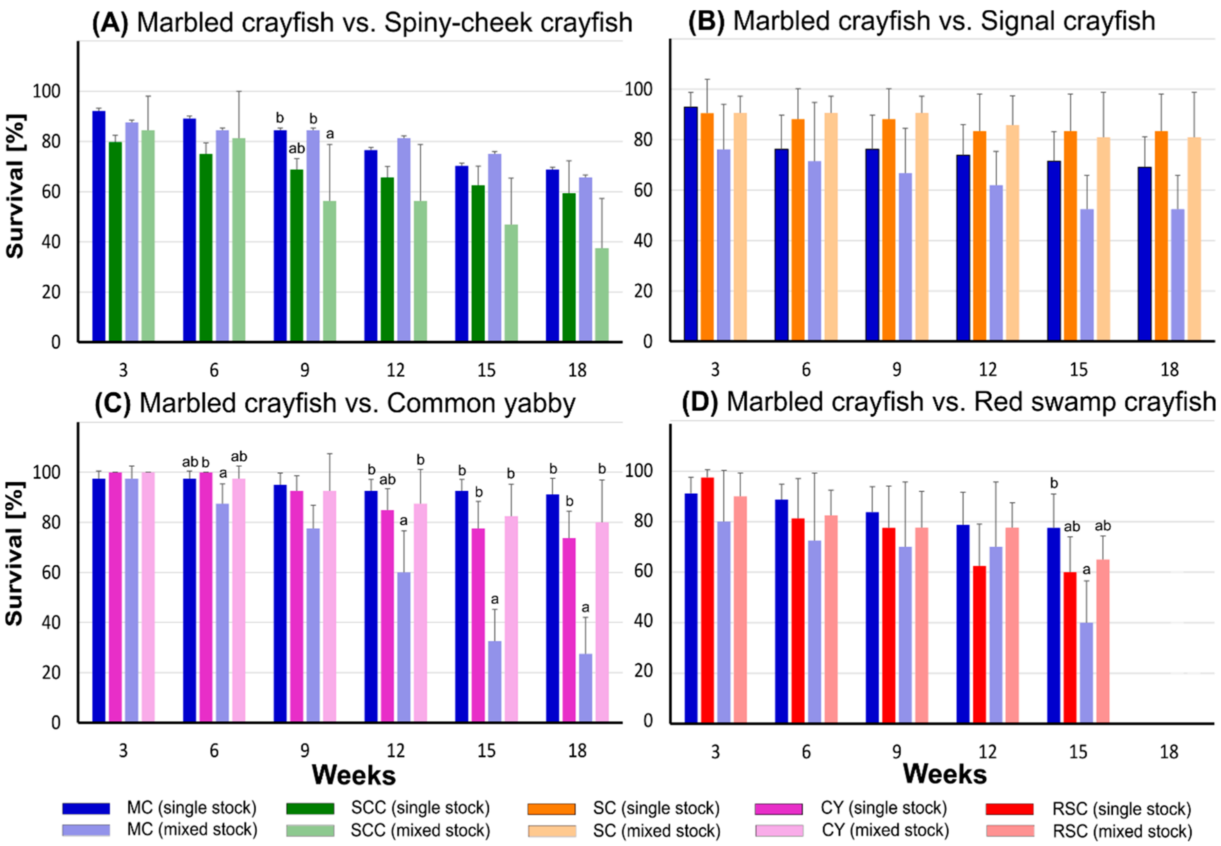

Figure S1. Survival (%) of marbled crayfish – MC in trials with spiny-cheek crayfish – SCC (A), signal crayfish – SC (B), common yabby – CY (C), and red swamp crayfish – RSC (D) kept in single-species and mixed stocks through the experiment (note darker and lighter colors used for single and mixed stocks, respectively).
